# Relationship between the earlobe crease and brain white matter abnormalities in apparently healthy subjects*

**DOI:** 10.3906/sag-1812-124

**Published:** 2019-04-18

**Authors:** Hatice Ayça ATA KORKMAZ

**Affiliations:** 1 Department of Radiology, Kanuni Research and Education Hospital, Trabzon Turkey

**Keywords:** Ear lobe crease, brain aging, white matter lesions, Fazekas scale

## Abstract

**Background/aim:**

In the present study we aimed to investigate whether the earlobe crease (ELC) might provide predictive information about white matter intensities (WMIs) in the brain that reflect brain aging.

**Materials and methods:**

A total of 350 individuals examined from January 2016 to July 2016 were screened. Patients with known demyelinating white matter disease, neurodegenerative disorders, cerebrovascular event history, or brain tumors were excluded from the study. Finally, 285 cases were included in the study. The four-point cerebral intensity classification system of Fazekas was used in the evaluation of the brain. The ELC was evaluated by inspection.

**Results:**

A total of 285 patients were enrolled consecutively. The incidence of WMI was significantly higher in patients with ELC than the others. Age (95% CI: 1.105–1.213, P < 0.001) and ELC (95% CI: 0.098–0.783, P = 0.015) were found as an independent determinants of abnormal WMI. ELC predicted abnormal WMIs with 89% specificity and 62% sensitivity.

**Conclusion:**

The presence of an ELC may provide predictive information in terms of detecting abnormal WMIs with prognostic impact in apparently healthy subjects.

## 1. Introduction

White matter intensities (WMIs) are generally discovered incidentally but are more prevalent with age, hypertension, and other cerebrovascular risk factors (1–3). WMIs are thought to be heterogeneous, such as in ischaemic small vessel disease (4). White matter injury lesions are associated with an increased risk of stroke (5), gait disturbance (6), cognitive impairment and decline (7), and dementia (8). In addition, white matter lesions from small vessel disease are associated with a regional pattern of gray matter atrophy that is reduced by blood pressure control, increased by aging, and linked with cognitive performance. In this regard, early detection of subtle WMIs might be important in terms of diagnosis and treatment, as some pharmacological and behavioral changes may delay the progression of WMI. WMIs are associated with cerebral ischemic white matter disease.

**Figure 1:**
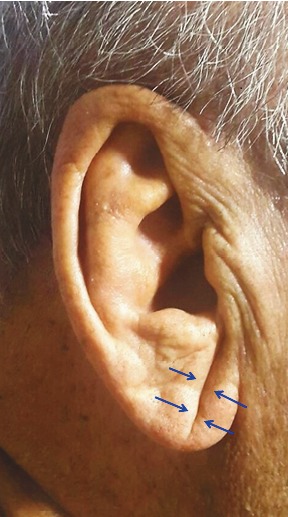
Earlobe crease (blue arrows).

 WMIs are related to ischemic white matter disorders affecting the same region of the brain. The progression of small vessel disease is common and more extensive in patients with cardiovascular or cerebrovascular diseases and atherosclerotic risk factors. Cardiovascular risk factors are related to cerebrovascular morphology and WMIs (9). The skin fold from the tragus to the auricle is called the earlobe crease (ELC) (Figure 1). Several studies have demonstrated an association between ELCs and systemic atherosclerosis. As there is a close association between the ELC and atherosclerosis, we hypothesized that we could obtain information related to brain aging simply by observing the presence of the ELC.

## 2. Materials and methods

### 2.1. Study population

All patients referred to our department of neurology with headache or cognitive deterioration were consecutively included in the study.

Patients with known demyelinating white matter disease, neurodegenerative disorders, cerebrovascular event history, or cerebral malignancies were excluded. 

This study was approved by the local ethics committee (2015/47) with written informed consent as acquired. This prospective study was designed according to the Helsinki Declaration and good clinical study guidelines. The individuals were examined with a 1.5 T magnetic resonance scanner (Siemens MAGNETOM Aera) in the department of radiology of a university hospital with routine clinical indications.

A radiologist with more than 15 years of experience in neuroradiology performed all investigations. The radiologist was blinded to the clinical inspection findings, laboratory results, early clinically suspected differential diagnoses, and final diagnoses of the patients.

A total of 350 subjects were evaluated in our MRI department from January 2016 to July 2016. Images from 17 patients were not obtained in sufficient quality because of patient movement. These 17 patients and 48 patients who did not want to participate in the study were excluded. Finally, 285 cases were included and 58 of these were evaluated as ELC-positive.

A survey questionnaire was filled out by all participants after approval of the information form. First questions were asked about age, sex, hypertension, diabetes mellitus, and smoking; then the presence of neurological complaints, such as headache, dizziness, walking imbalance, sudden neurological complaints, and double vision, was addressed; and finally the presence of the ELC was evaluated by inspection.

### 2.2. Magnetic resonance imaging analysis

The standard MRI protocol for neuroimaging was as follows: T2-weighted axial plane, voxel size (VS) 0.9 mm × 1.12 mm, slice thickness (ST) 3 mm, interslice distance (ISD) 0.3 mm, TE 110 ms, TR 4250 ms, flip angle 90°, fluid attenuated inversion recovery (FLAIR); axial plane, sagittal plane, VS 0.9 mm × 1.12 mm, ST 4 mm, ISD 0.4 mm, TE 100 ms, TR 4000 ms, T1-weighted VS 0.94 mm × 1.13 mm × 2 mm, TE 4.6 ms, TR 9.1 ms, axial plane, VS 2.05 mm × 2.56 mm, ST 5 mm, ISD 1 mm, TE 25 ms, TR 700 ms, flip angle 90°. All images were stored in our hospital’s PACS DATA system.

The MRI studies were evaluated by a radiologist with more than 15 years of neuroradiology experience. The Fazekas scale (10) was used to evaluate WMIs, especially on T2W FLAIR sequences, as an indicator of ischemic small vessel disease and brain aging.

The T1WI and T2WI sequences were evaluated. T1WI is commonly used to evaluate anatomy and T2WI is used for additional pathological findings.

### 2.3. Cerebral hyperintensity classification system of Fazekas

**Figure 2:**
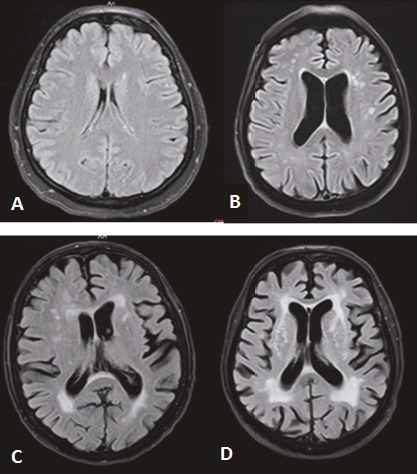
Fazekas classification system was demonstrated by MR in FLAIR sequence. A) Fazekas 0: normal white matter intensity, none or a single punctate lesion. B) Fazekas 1: multiple punctate lesions. C) Fazekas 2: beginning of confluency of lesions. D) Fazekas 3: large confluent lesions.

The level of WMI was scored on the special cerebral hyperintensity classification system of Fazekas with four-point scale. The classification was evaluated on MRI images with the T1W, T2W, and FLAIR sequences. Images from Fazekas 0–3 scale examples are demonstrated in Figure 2.

WMIs on MRI were described as indistinct hyperintense foci of 5 mm on both transverse T2 and FLAIR images. Ischemic lacunae were well-defined regions of hyperintense focuses of 2 mm on T2 as well as similar intensity of cerebrospinal fluid. Foci with similar size and appearance were considered as perivascular spaces. Nevertheless, perivascular spaces neighboring the anterior commissure may be larger. Basal ganglia lesions were evaluated similarly to white matter lesions, even those located in the gray matter.

WMIs and lacuna observed on MRI in the elderly population are often regarded as a sign of ischemic small vessel disease. The overall degree of WMI in the brain can be assessed with the Fazekas scale. We used a four-point Fazekas scale scoring system to classify the WMIs. Fazekas score of 0 indicated normal white matter intensity without WMI, Fazekas 1 (F1) indicated multiple WMIs, Fazekas 2 (F2) indicated WMIs tending towards bridging, and Fazekas 3 (F3) indicated large, intermingled, and fused WMIs.

F1 is accepted as normal in older adults. F2 and F3 are considered pathological, but may be seen in cases with normal intellect and function. On the other hand, this was a high-risk population for intellectual and functional loss, as subjects in the Fazekas 3 group have a 25% loss of function within 1 year (11). Three-year follow-up showed rapid functional loss in these patients with severe white matter lesions (12).

### 2.4. Statistical analysis

Continuous variables are presented as mean ± standard deviation, and categorical variables are presented as percentages. 

The differences between the categorical variables between the groups were evaluated by chi-square test. P < 0.05 was considered significant. Sensitivity and specificity values were calculated when a significant threshold was found. Statistical analysis was performed with SPSS 22.0 (13).

## 3. Results

**Figure 3:**
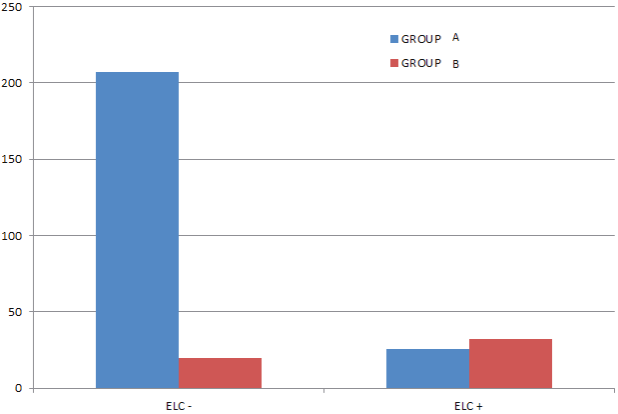
Group A (blue graphic) refers to healthy individuals without significant WMI (Fazekas 0); group B (red graphic) refers to the individuals with WMIs (Fazekas 1, 2, and 3). WMI: White matter intensity, ELC: earlobe crease.

Analysis of the study population is presented in Table 1. Mean age was 58 ± 16 years. The ELC was found in 58 (20%) patients. A total of 233 (81%) patients had normal WMI compared to 52 (19%) patients who had abnormal WMI. Two groups were created from among the patients: group A had normal WMIs (Fazekas 0), and group B had abnormal WMIs (Fazekas score of ≥1). Age, presence of the ELC, and hypertension differed significantly between the two groups (Table 2). The frequency of WMI according to the incidence of the ELC is demonstrated in Figure 3.

**Table 1:** Analysis of the study population.

Age, years	59 ± 11
Males, n (%)	72 (25%)
Hypertension, n (%)	66 (23%)
Diabetes, n (%)	24 (8%)
Smoking, n (%)	76 (27%)
Presence of ELC, n (%)	58 (20%)
Fazekas *(-)	233 (82%)
Fazekas **(+)	52 (18%)

ELC: Earlobe crease. *(-): Patients with normal WMI (Fazekas 0). ** (+): Patients with abnormal WMI (Fazekas ≥1).

**Table 2:** Analysis of the study population according to WMI.

Variables	Fazekas *(-)	Fazekas **(+)	P-value
Age, years	48 ± 10	66 ± 10	<0.001
Males, n (%)	44 (20%)	28 (12%)	0.11
Hypertension	46 (20%)	20 (38%)	0.04
Diabetes	19 (8%)	5 (9%)	0.73
Smoking	64 (28%)	12 (23%)	0.51
Presence of ELC, n (%)	26 (11%)	32 (62%)	<0.001

WMI: White matter intensity, ELC: earlobe crease. *(-): Patients with normal WMI (Fazekas 0). **(+): Patients with abnormal WMI (Fazekas ≥1).

Binary logistic regression analysis was performed to identify the independent determinants of abnormal WMI (Table 3). It demonstrated that age (95% confidence interval [CI]: 1.105–1.213, P < 0.001) and the ELC (95% CI: 0.098–0.783, P = 0.015) were independent determinants of the existence of abnormal WMI.

**Table 3:** Binary logistic regression analysis for independent determinant of Fazekas score.

	P-value	95% CI
Age, years	<0.001	1.105–1.213
Hypertension	0.226	0.195–1.472
Presence of ELC	0.015	0.098–0.783

ELC: Earlobe crease.

We also calculated the specificity and sensitivity of the ELC in terms of diagnosing abnormal WMI. The ELC predicted abnormal WMI with 89% specificity and 62% sensitivity.

## 4. Discussion

In the present study, we have found a significant relationship between ELC and asymptomatic WMI. The ELC may be useful to predict WMI abnormalities with high sensitivity and specificity in an apparently healthy population.

The incidence of cognitive impairment is increasing with aging, and its social, economic, and emotional burden is immense (14). Understanding the etiopathogenesis of impaired consciousness associated with aging can prevent this condition and protect cognitive abilities. Brain lesions due to ischemic small vessel disease and especially WMI have independently been related with cognitive impairment. Evidence suggests that white matter changes have a significant impact on daily activities, including gait and executive functioning.

 Silent brain infarction, i.e. WMI and lacunae viewed as cerebral small vessel disease, is being reported more frequently in older people with the development of medical technology (15). The Rotterdam MRI Scan Study showed that newly developed silent brain infarcts are associated with the presence of cardiovascular risk factors, such as hypertension, dyslipidemia, diabetes, carotid plaques, smoking, and age (15). Several reports revealed that the increasing severity of WMI and lacunae is related to a decline in cognitive function scores in these patients (15). Risks for cardiovascular disease and the development of cognitive impairment are the same. Cerebrovascular disease is considered an important contributing factor to vascular cognitive impairment. Furthermore, silent brain infarction provoked by small vessel disease, rather than large cortical cerebral infarction, is more considerable in the occurrence of vascular cognitive decline, although various cardiovascular risk factors are related with the development of both types of cerebral infarction.

The relationship between cerebrovascular events and CAD suggests that brain and cardiovascular diseases may have similar etiopathogenesis, although both are strongly associated with advanced age.

There is little known in terms of the possible etiopathogenesis producing ischemic small vascular disease in the brain and injury of CAD. However, the etiopathogenesis of both disease groups is closely related to atherosclerosis due to hypertension, endothelial damage, and local inflammatory events.

The possible relationship between the ELC and CAD was first reported by Frank in 1973 (16). A recent review of the skin manifestations of atherosclerosis suggests that ELC may be an early marker of atherosclerosis (17). A metaanalysis suggested that ELC may be an early sign of CAD with 67% specificity and 62% sensitivity (18). 

In spite of these results, no certain statement of etiopathogenesis was determined between ELC and CAD.

The myocardium and ELC have a common genetic microvascular origin (19) and therefore the ELC and CAD are thought to be formed by a common pathophysiological process due to atherosclerosis. 

In addition, the ELC is significantly associated with increased arterial stiffness and carotid intima media thickness, which are considered surrogate markers of atherosclerosis.

Several limitations to our study should be mentioned. The size of the study was relatively small and it may not reflect the general population, as we did not include patients with overt cerebrovascular disease. The clinical significance of the WMIs found in our study was not established because of the absence of long-term follow-up. We also did not grade WMI abnormalities because only a few patients had a Fazekas score of ≥3. We attribute this to the study protocol, whereby we included only asymptomatic patients. Finally, our study design was cross-sectional; therefore, it did not provide any details related to the causative mechanisms of the association between the ELC and WMI.

In conclusion, although the etiopathogenesis of the relation between the ELC and WMI remains to be clarified, this is the first study to report a highly positive association between the ELC and WMI in a large group of participants. Physicians could obtain valuable information related to the white matter of the brain by observing the ELC during clinical examinations.
